# Supplementary material for: Metabolomics Reveals Heterogeneity in the Chemical Composition of Green and White Spears of Asparagus (A. officinalis)
Source: Metabolites. 2021 Oct 16;11(10):708. doi: 10.3390/metabo11100708 (PMC8538002; doi:10.3390/metabo11100708)
Supplement: Supplementary file 1 [file metabolites-1399415_Suuplementary Figures_Rebuttal.pdf › metabolites-1399415_Suuplementary Figures_Rebuttal.pdf]

Article

Metabolomics reveals heterogeneity in the chemical composition of green and white spears of asparagus (*A. officinalis*)

Eirini Pegiou [1], Qingrui Zhu [1,2] , Paraskevas Pegios [3], Ric C. H. de Vos [4], Roland Mumm [4] and Robert D. Hall [1,4,*]

Supplementary Figures

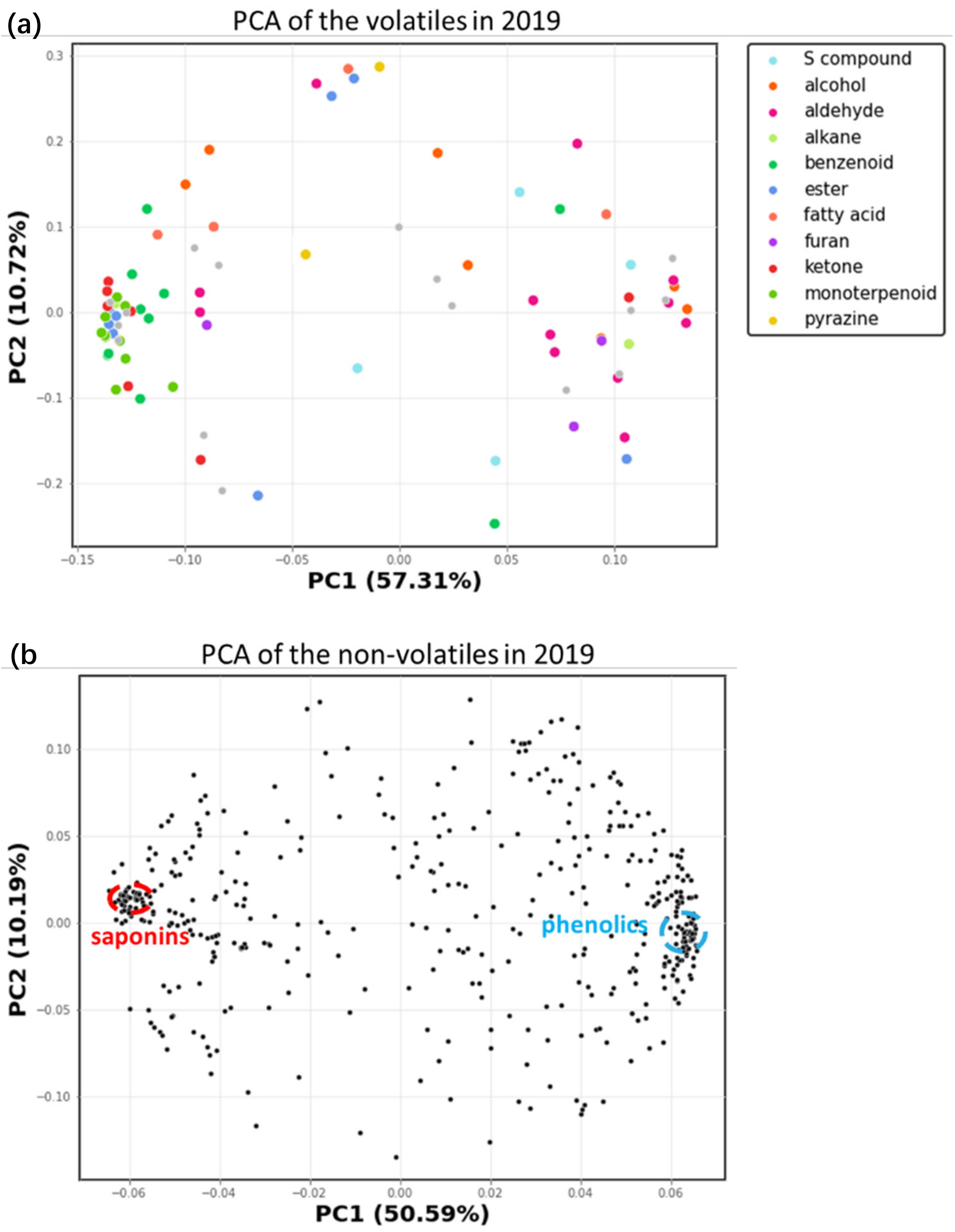

Figure S1. PCA of the GC-MS and LC-MS data of the green and white asparagus spears harvested in 2019. The first two PCs are presented and the explained variance is shown in parenthesis on the axes: **(a)** Loading plot of the volatile profiles. Colours indicate the compound class of the identified volatiles. Unidentified volatiles are coloured grey; **(b)** Loading plot of the non-volatile profiles. All non-volatiles are colored black except for identified saponins and polyphenols which indicated by the red and blue circles respectively.

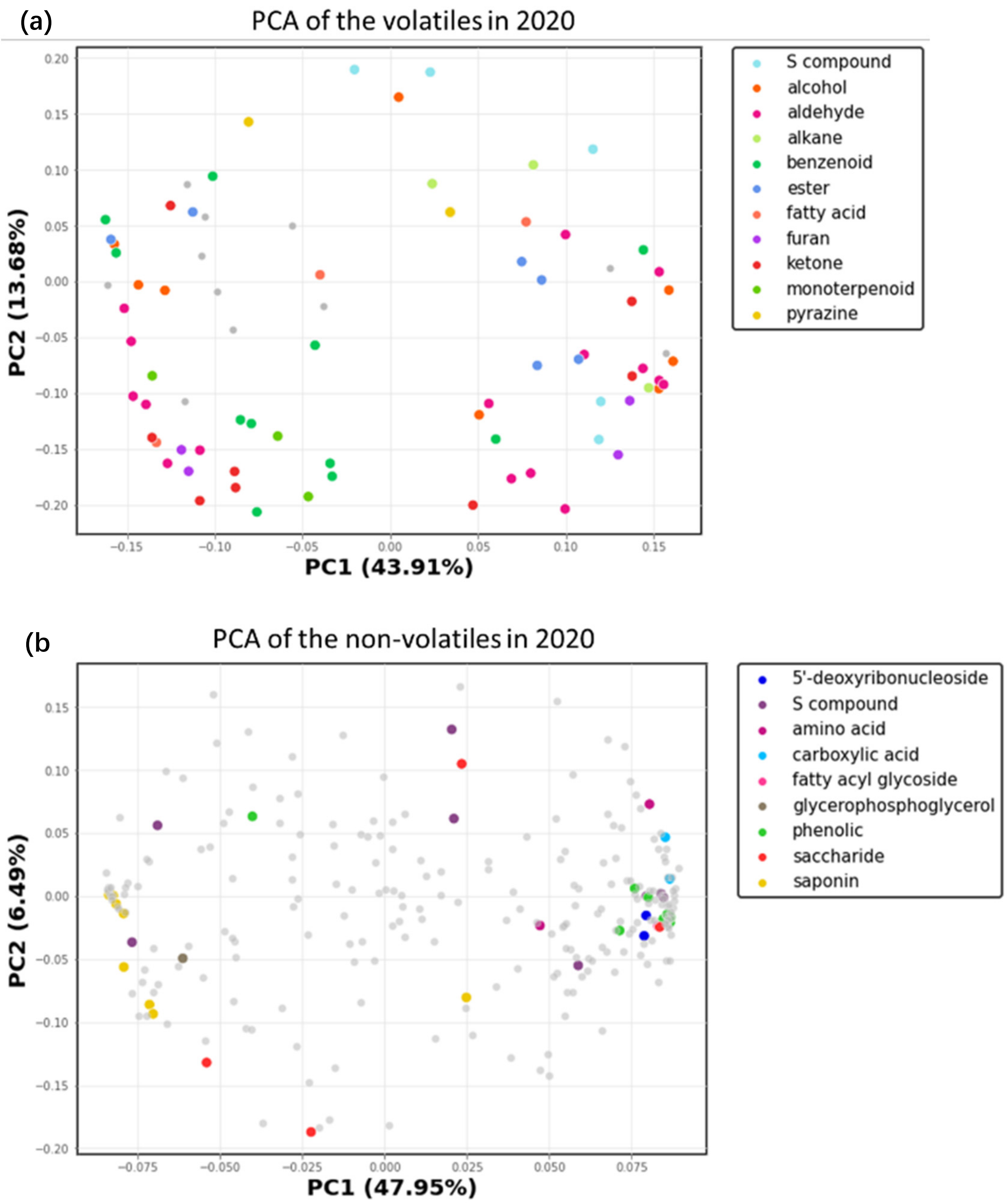

Figure S2. PCA of the GC-MS and LC-MS data of the green and white asparagus spears harvested in 2020. The first two PCs are given and the explained variance is shown in parenthesis on the plots axes. Colours indicates compound class of the identified metabolites. Unidentified compounds are coloured grey: **(a)** Loading plot of the volatile profiles; **(b)** Loading plot of the non-volatile profiles.

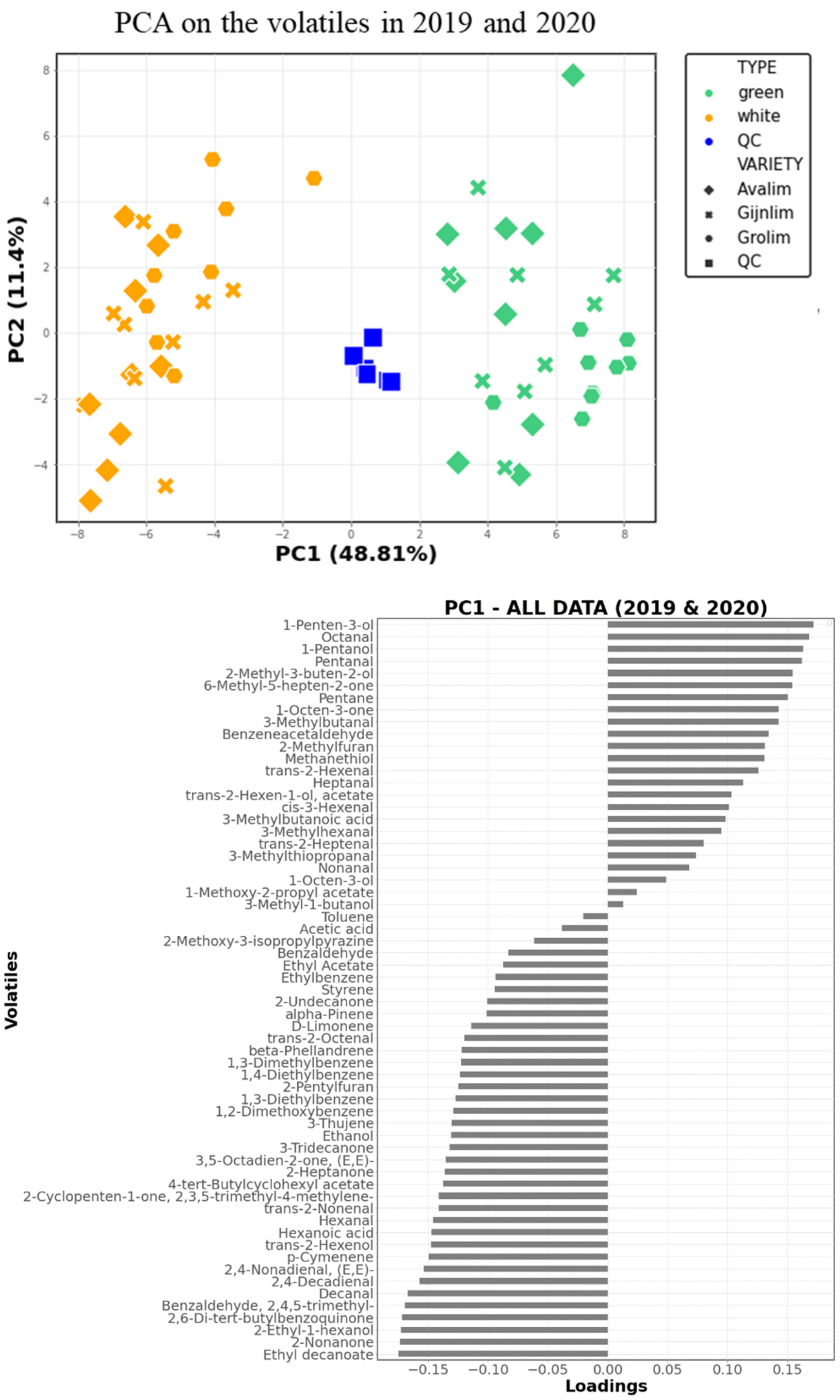

Figure S3. PCA of the 61 identified volatiles detected in the green and white asparagus spears harvested and analysed in 2019 and 2020. Data were log-transformed and autoscaled before fusing them: **(a)** Score plot of the first two PCs where the explained variance is shown in parenthesis on the plots axes. Colours indicate the asparagus type, symbol shapes indicate asparagus variety; **(b)** Loadings of the first PC sorted in descending order.

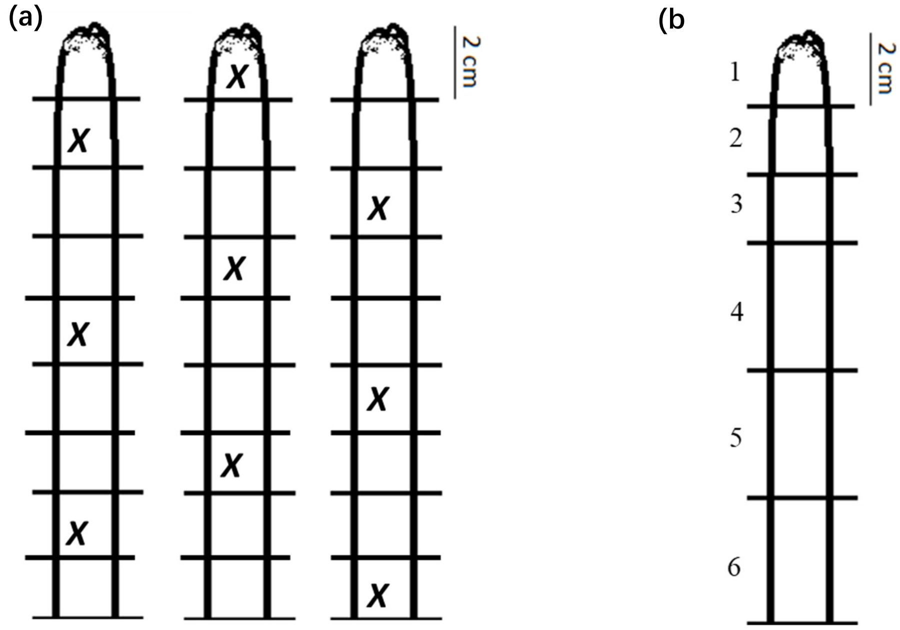

Figure S4. Schematic representation of the composition of a pooled sample produced for the analysis of the asparagus varieties. For each pooled sample of three spears, the shoots were cut into 2 cm sections: **(a)** One piece per section (X) was taken across three spears to generate a pooled biological replicate sample representative of a whole spear; **(b)** For the spatial metabolomics pilot, the equivalent pieces from three spears were collected to make pooled samples per section. The numbers given in (b) correspond to the annotations in Figure 5; For green spears, 5 sections are shown as green shoots were shorter than white asparagus (Figure 1).
